# 76644 Cognitive and Behavioral Outcomes in Adolescents with Sickle Cell Disease Before and After a Telehealth Cognitive Remediation Program to Prepare for Transition of Care

**DOI:** 10.1017/cts.2021.704

**Published:** 2021-03-30

**Authors:** Donna Murdaugh, Tiffany Tucker, Victoria Seghatol-Eslami, Anne Stewart, Jeffery Lebensburger, Eric Wallace, Smita Bhatia

**Affiliations:** University of Alabama at Birmingham School of Medicine

## Abstract

ABSTRACT IMPACT: This study is providing a telehealth intervention for the first time in patients with sickle cell disease with the goal of improving cognitive functioning and preparing adolescents for successful transition of care to adult healthcare providers. OBJECTIVES/GOALS: There is a high prevalence of cognitive impairment in adolescents with sickle cell disease (SCD). The purpose of this study is to test the efficacy of an individualized cognitive remediation program designed to promote not just cognitive function, but also adaptive and self-management skills necessary for successful transition to independence. METHODS/STUDY POPULATION: 12 participants with SCD (5 males, ages 10-16) participated in an individualized program, Cognitive-Remediation of Executive and Adaptive Deficits in Youth [C-READY], consisting of three main components: individual goal-based therapy, parent training sessions, and home skill practice. C-READY sessions occur one-on-one with a trained therapist for 8 sessions conducted over 4 weeks. Weekly parent training sessions are also conducted as part of the C-READY program. All of these sessions occurred via telehealth video-calling between the therapist and the adolescent/parent. Participants were evaluated before and after the C-READY program using neuropsychological assessment measures and transition readiness questionnaires. Parents also completed ratings on telehealth delivery, content, and timing. RESULTS/ANTICIPATED RESULTS: Repeated measures ANOVA indicated significant improvement in transition readiness behaviors as rated by parents, including improved independence in medication management (p = 0.029) and in talking with their healthcare providers (p = 0.019). Significant improvement was also demonstrated on a neuropsychological measure related to executive function skills, specifically inhibition and switching (p = 0.012). Results from telehealth surveys (rated on a 5-point Likert scale) indicated overall satisfaction with services (4.2/5), including visual (4/5) and voice quality (4.3/5) of telehealth equipment. Ratings also indicated feeling that their privacy was respected (4/5) and that their interactions with their therapist were appropriate and sensitive (4.5/5). DISCUSSION/SIGNIFICANCE OF FINDINGS: These results provide support for interventions that focus on cognitive skills to improve behaviors necessary for successful transition of care in youth with SCD. Results are also promising for delivery via telehealth in order to address barriers related to access to care. Future results will continue to be reported, as this study is currently ongoing.

